# Supplementary material for: A universal molecular control for DNA, mRNA and protein expression
Source: Nat Commun. 2024 Mar 20;15:2480. doi: 10.1038/s41467-024-46456-9 (PMC10954659; doi:10.1038/s41467-024-46456-9)
Supplement: Supplementary file 1 — Supplementary Information [file 41467_2024_46456_MOESM1_ESM.pdf]

A universal molecular control for DNA, mRNA and protein expression.

Helen M. Gunter[1,2,3], Scott Youlten[4,5,6], Andre L. M. Reis[7,8,9], Tim McCubbin[1,3], Bindu Swapna Madala[5,8], Ted Wong[5], Igor Stevanovski[7,8], Arcadi Cipponi[5,6], Ira W. Deveson[7,8,9], Nadia S. Santini[10], Sarah Kummerfield[5,6], Peter Croucher[5,6], Esteban Marcellin[1,3] & Tim R. Mercer[1,2,3,5,*]

[1] Australian Institute of Bioengineering and Nanotechnology, University of Queensland, Brisbane, Queensland, Australia.

[2] BASE mRNA Facility, University of Queensland, Brisbane, Queensland, Australia.

[3] ARC Centre of Excellence in Synthetic Biology, The University of Queensland, Queensland, Australia.

[4] Department of Genetics, Yale University School of Medicine, New Haven, CT 06510, USA.

[5] Garvan Institute of Medical Research, Sydney, New South Wales, Australia.

[6] St Vincent's Clinical School, University of New South Wales, Sydney, New South Wales, Australia.

[7] Genomics and Inherited Disease Program, Garvan Institute of Medical Research, Sydney, New South Wales, Australia.

[8] Centre for Population Genomics, Garvan Institute of Medical Research and Murdoch Children's Research Institute, Sydney, New South Wales, Australia.

[9] School of Electrical and Information Engineering, University of Sydney, Sydney, New South Wales, Australia.

[10] Centro Nacional de Investigación Disciplinaria en Conservación y Mejoramiento de Ecosistemas Forestales, INIFAP, Ciudad de México 04010, Mexico.

[*] Corresponding Author t.mercer@uq.edu.au

SUPPLEMENTARY FIGURES 1-14

SUPPLEMENTARY FIGURE LEGENDS.

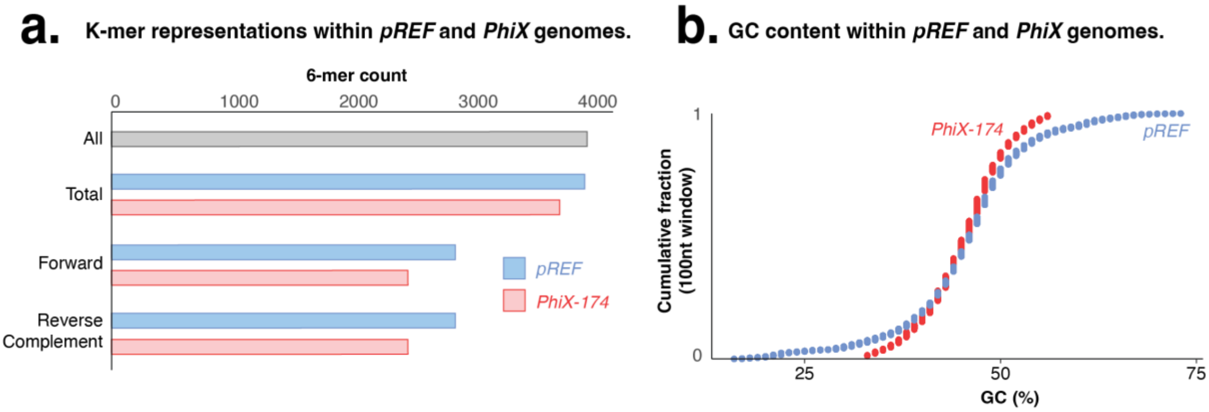

Supplementary Figure S1. Design of the synthetic control, *pREF*. (a) Representation of 6-mer sequences within *pREF* and the *phiX-174* genome. (b) Cumulative distribution of GC-content within 100nt sliding window across *pREF* and the *phiX-174* genome. Source data are provided in a Source Data File.

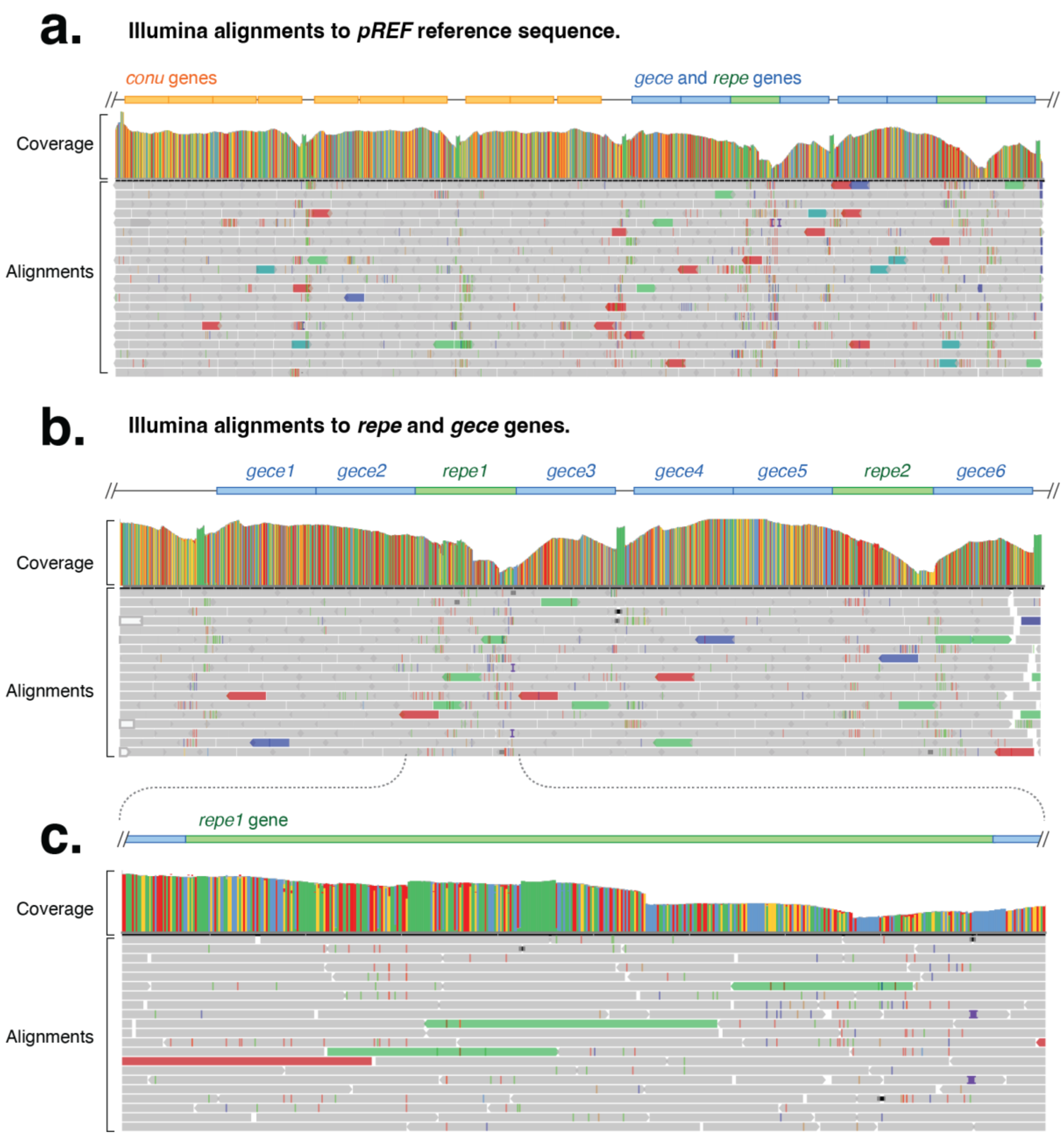

Supplementary Figure S2. **Genome-browser view of Illumina DNA sequencing alignments to *pREF* reference sequence. (a)** Alignment of Illumina paired-end reads to *pREF* reference sequence. **(b-c)** Detail shows alignments (and errors) at GC-rich and repetitive sequences in *gece* and *repe* genes. Source data are provided in a Source Data File.

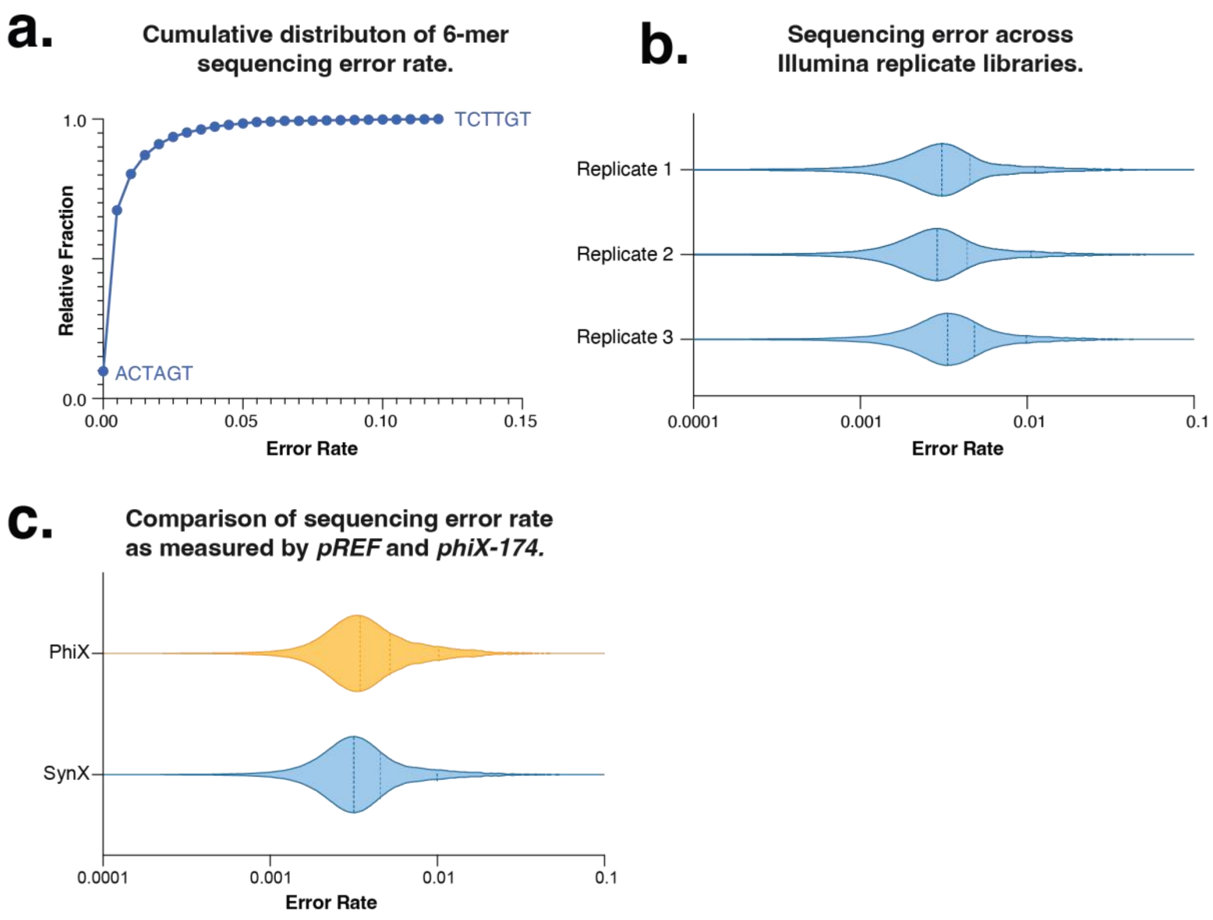

Supplementary Figure S3. Illumina pREF DNA sequencing performance. (a) Cumulative distribution of sequencing errors rate 6-mer in *pREF* sequences. (b) Violin-plot showing error-rate distribution as measured by *pREF* across three further replicate Illumina libraries. (c) Violin plot showing error-rate distribution as measured by *pREF* (blue) and *phiX-174* (yellow). Source data are provided in a Source Data File.

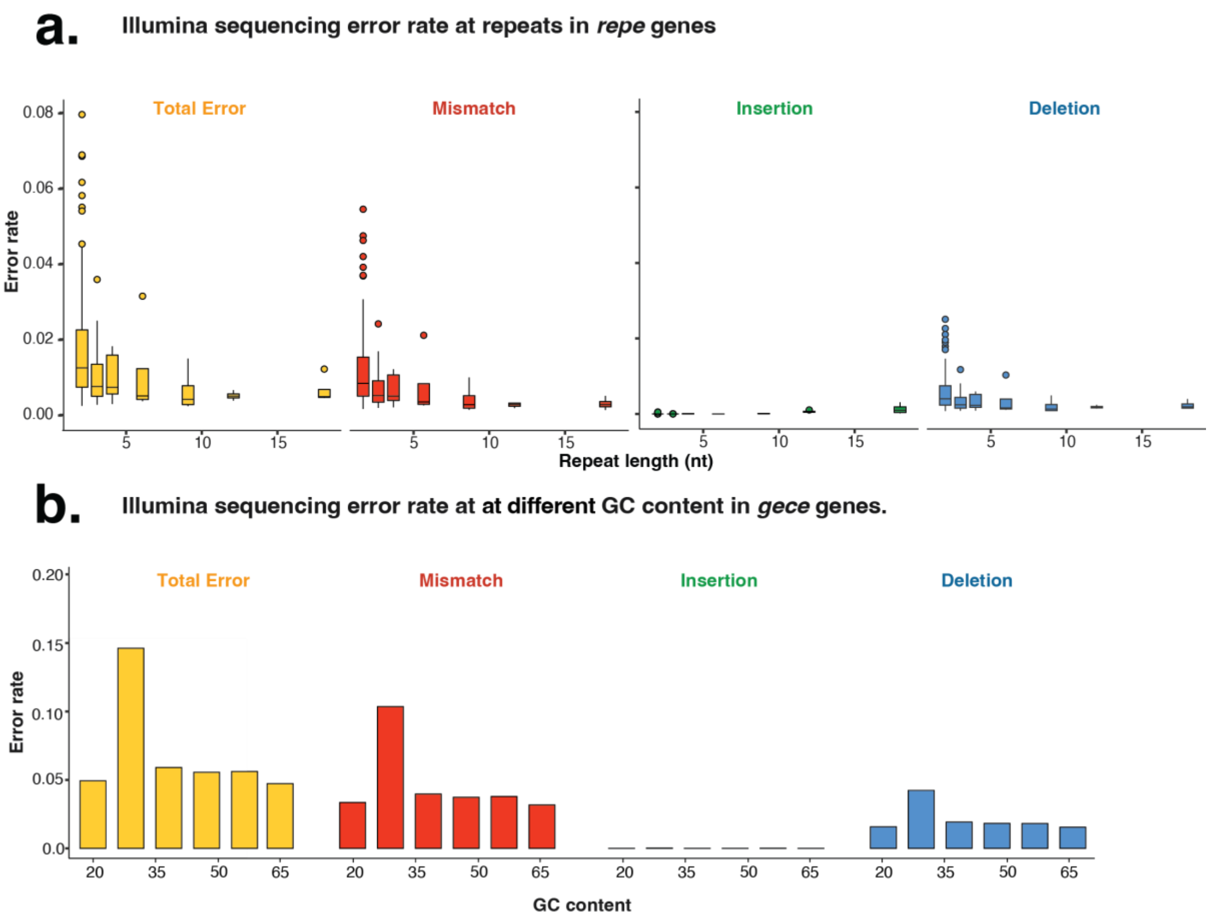

Supplementary Figure S4. Illumina DNA sequencing performance at difficult sequences. (a) Box-whisker plot shows error-rate at homopolymer repeats of increasing length in *repe* genes. Box plot extends from 25th to 75th percentiles, centre line is the median, and whiskers cover the 10th and 90th percentiles. Coloured circles are outliers (n=1 biologically independent sample). **(b)** Bar charts show mean Illumina sequencing error rates for six *gece* genes, which differ in their GC content (n=1 biologically independent sample). Source data are provided in a Source Data File.

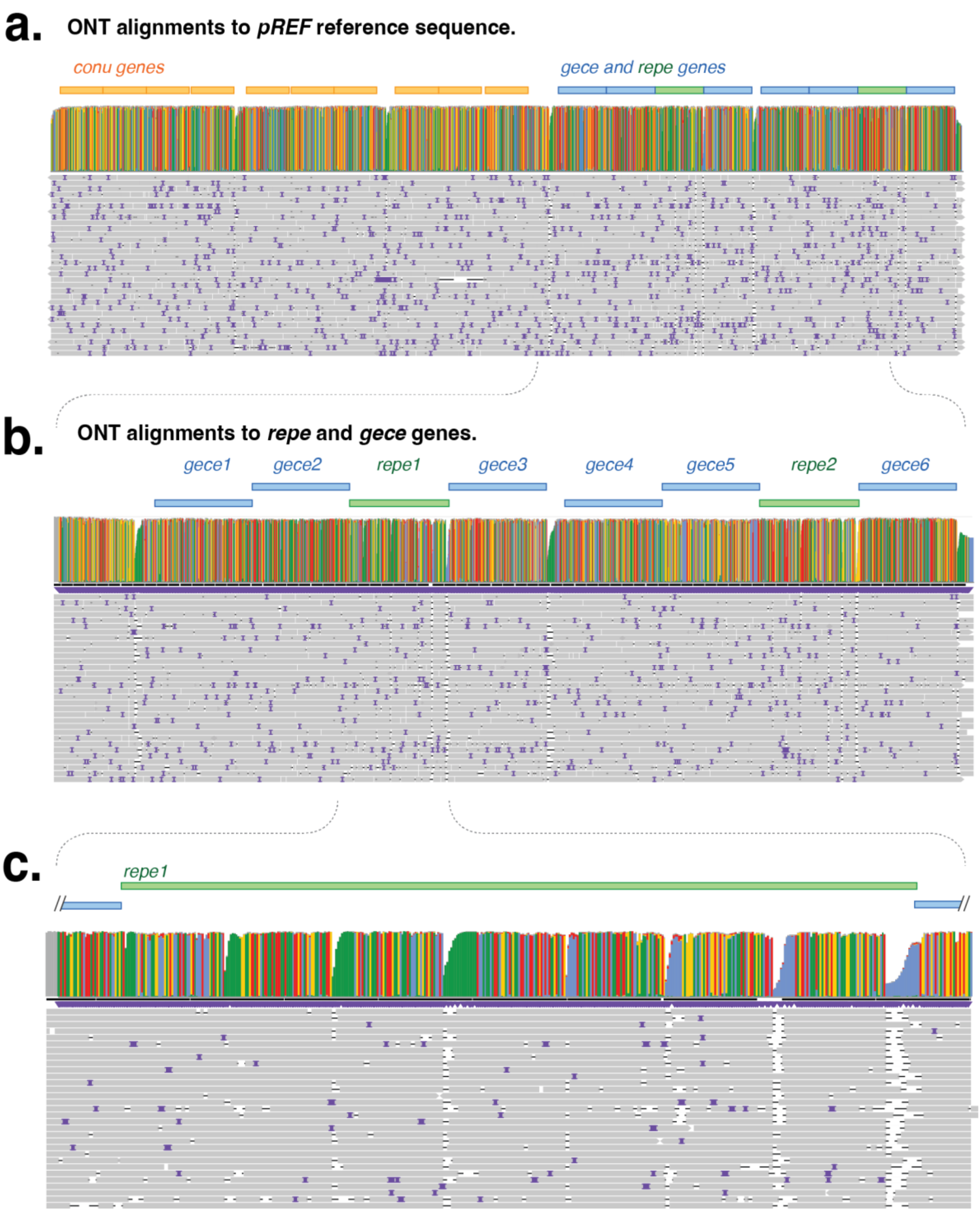

Supplementary Figure S5. **Genome-browser view of ONT DNA sequencing alignments to *pREF* reference sequence. (a)** Alignment of ONT reads to *pREF* reference sequence. **(b-c)** Detail shows alignments (and errors) at GC-rich and repetitive sequences in *gece* and *repe* genes. Source data are provided in a Source Data File.

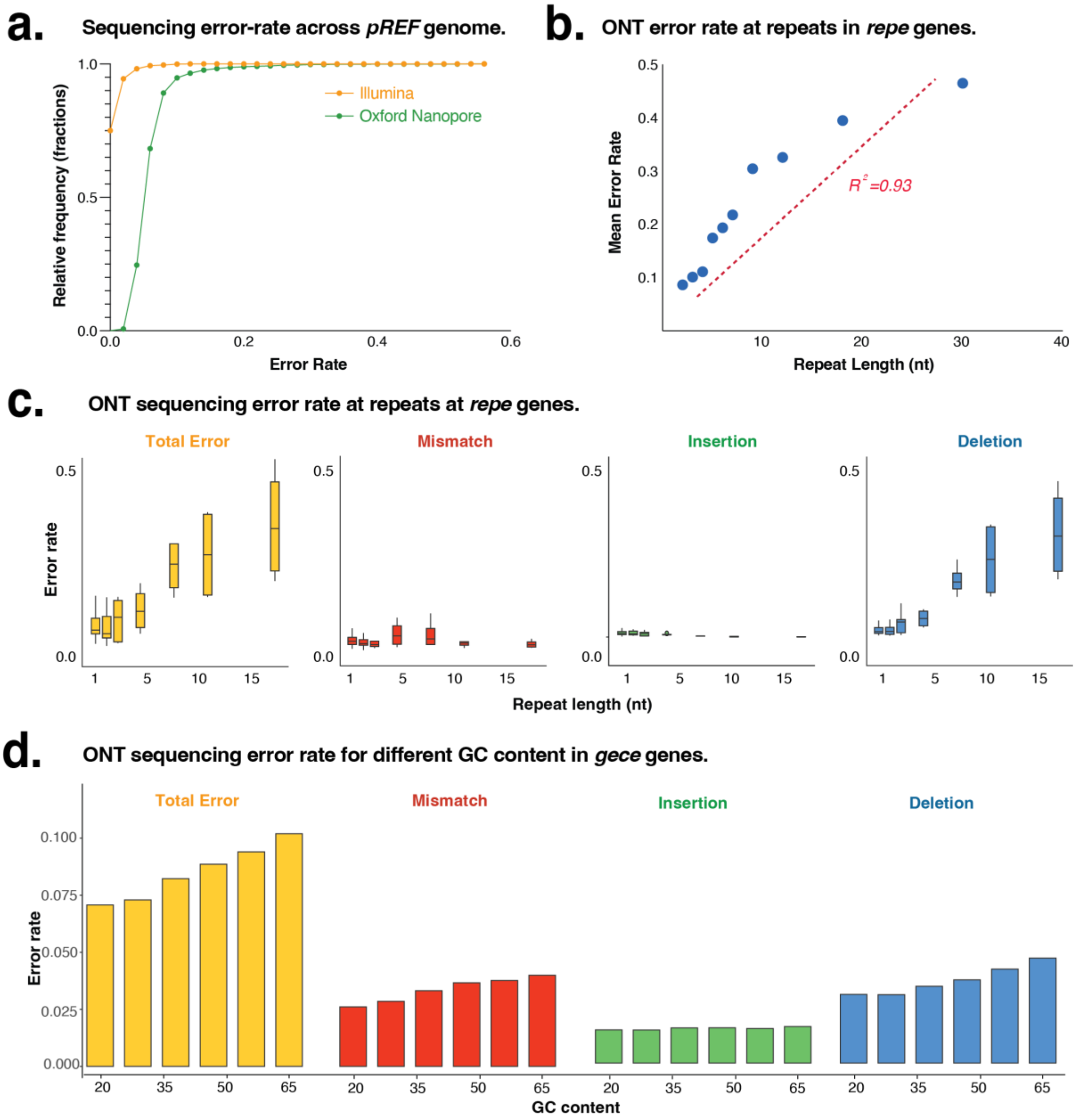

Supplementary Figure S6. ONT sequencing performance at repetitive and GC-rich genes. (a) Cumulative distribution of sequencing error-rate at 6-mers across *pREF*. **(b)** Scatter-plot shows the error-rate of ONT sequencing at repeats of increasing length in *repe* genes. **(c)** Box-whisker plot shows error-rate at homopolymer repeats of increasing length in *repe* genes. Box plot extends from 25th to 75th percentiles, centre line is the median, and whiskers cover the 10th and 90th percentiles (n=1 biologically independent sample). **(d)** Bar charts show mean ONT sequencing error rates for six *gece* genes, which differ in their GC content (n=1 biologically independent sample). Source data are provided in a Source Data File.

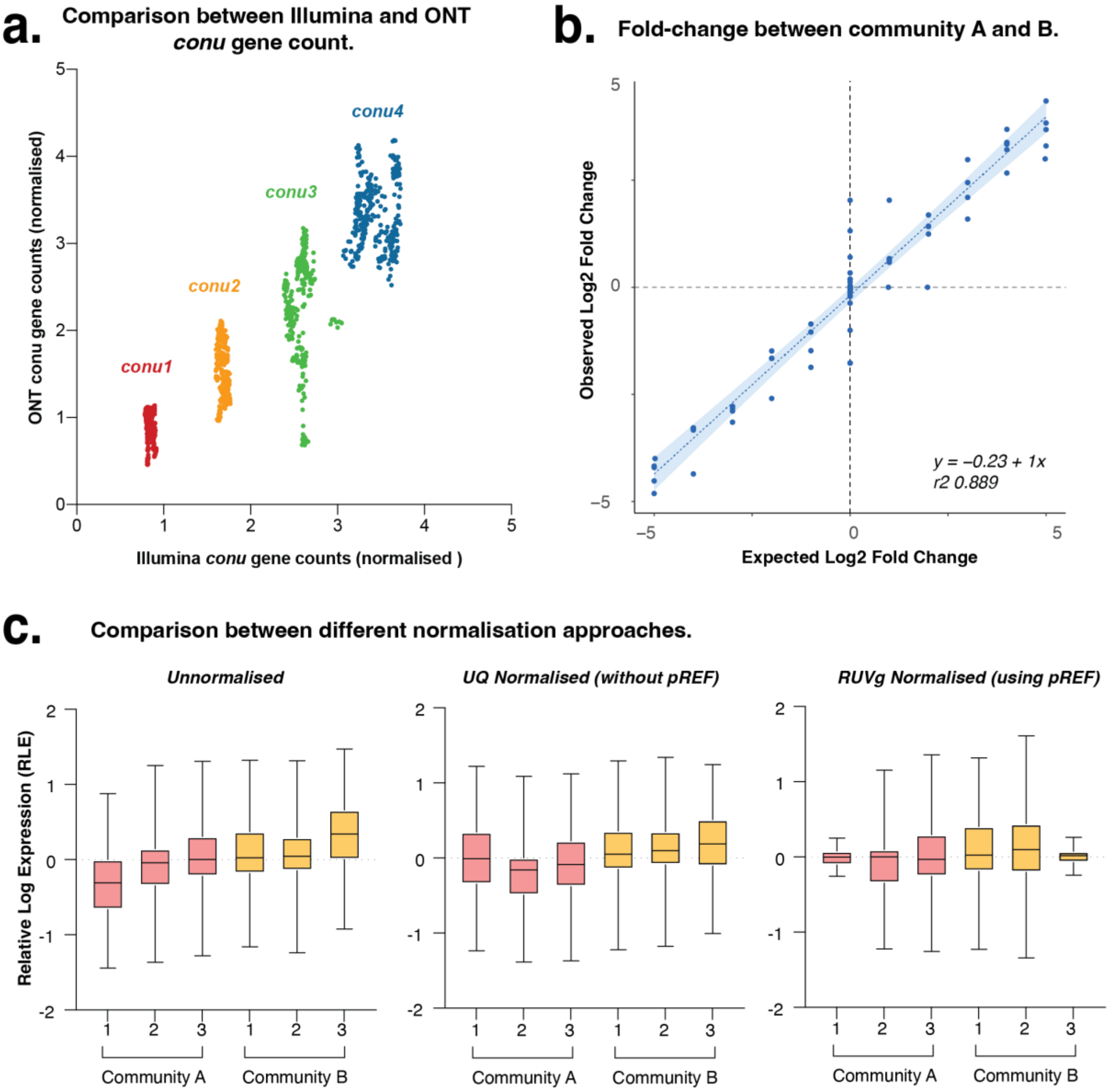

Supplementary Figure S7. Quantitative performance of NGS libraries as measured by *conu* genes. (a) Scatter-plot compares the measurement of normalised gene counts across 31-mer sliding windows for *conu1-4* genes, in Illumina and ONT sequencing libraries. **(b)** Scatter-plot shows compares the observed fold-change differences to expected fold-change differences between triplicate libraries prepared from mock microbial communities A and B. Dotted line is the slope of a linear regression, with 95% CI. **(c)** RLE plots demonstrate differences in abundance following no normalization, Upper Quartile normalisation (UQ) and RUVg normalization (using *pREF* to determine scaling factors). Box plot extends from 25th to 75th percentiles, centre line is the median, and whiskers cover the 10th and 90th percentiles (n=3 biologically independent samples). Source data are provided in a Source Data File.

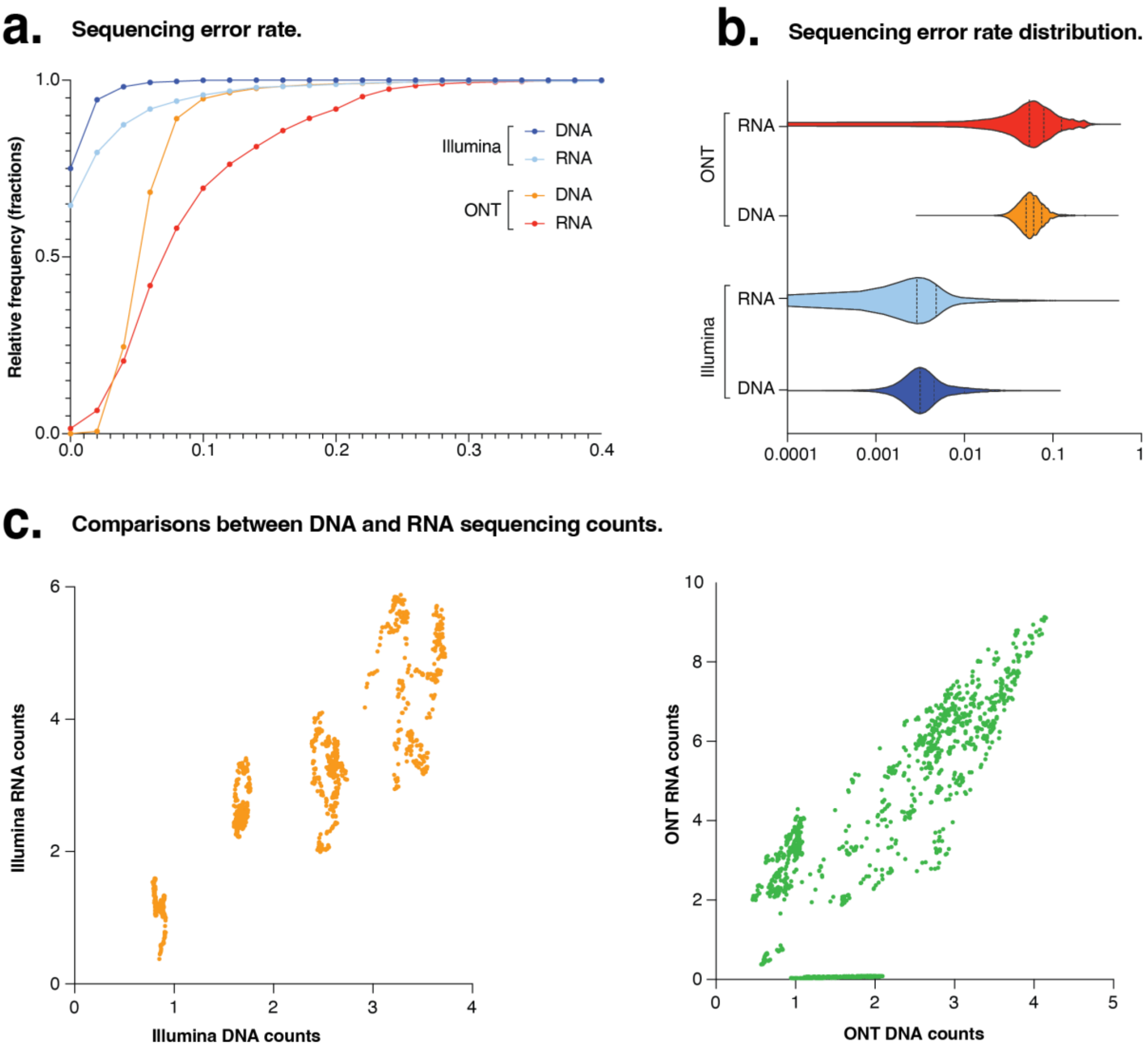

Supplementary Figure S8. RNA sequencing performance as measured using *pREF* mRNA controls. (a) Cumulative distribution of sequencing error rates across *pREF* 6-mers. **(b)** Violin-plot shows error profile distribution of Illumina and ONT RNA sequencing. (c) Scatter-plot shows ***conu*** read counts from RNA and DNA sequencing using both Illumina and ONT sequencing. Source data are provided in a Source Data File.

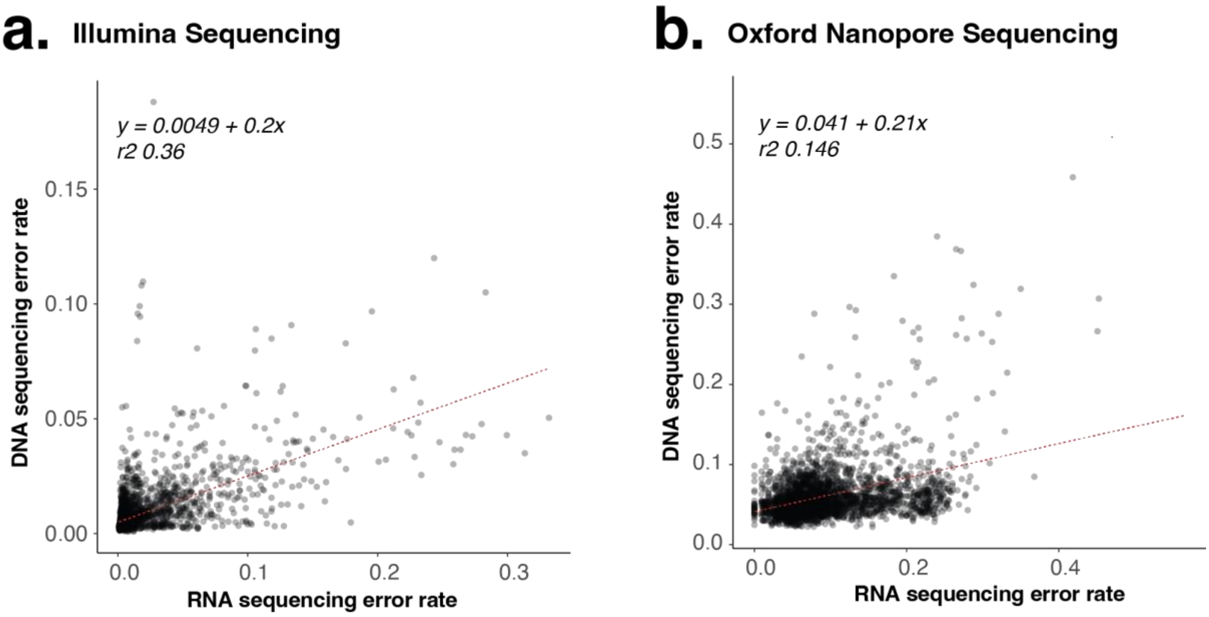

Supplementary Figure S9. Sequencing error across ranked k-mers. DNA sequencing error rate across ranked k-mers using **(a)** Illumina and **(b)** ONT sequencing. Source data are provided in a Source Data File.

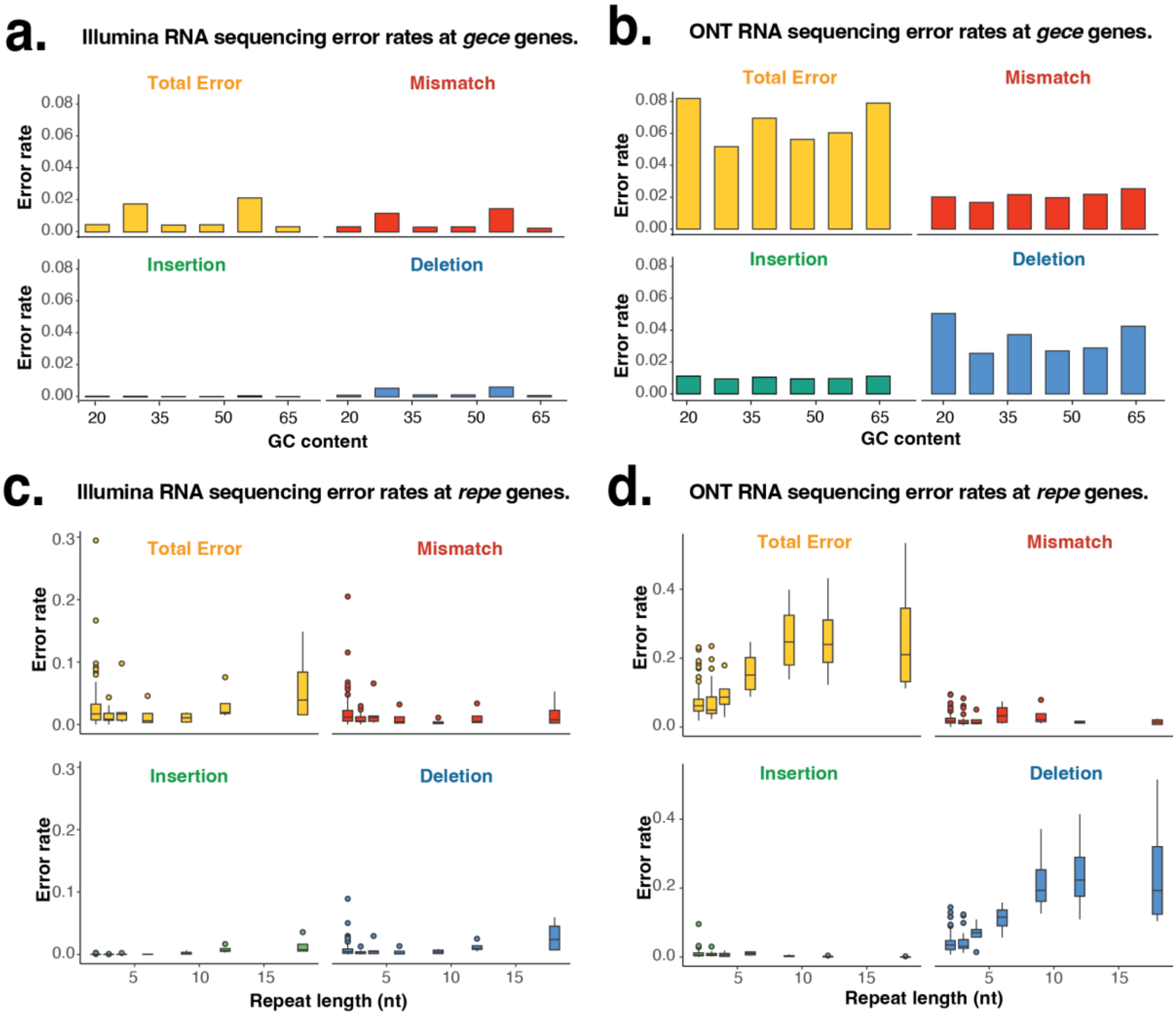

Supplementary Figure S10. RNA sequencing performance at repetitive and GC-rich genes. (a) Bar charts show mean Illumina sequencing error rates for six sp6 transcribed *gece* genes, which differ in their GC content (n=1 biologically independent sample). **(b)** Bar charts show mean ONT sequencing error rates for six sp6 transcribed *gece* genes, which differ in their GC content (n=1 biologically independent sample). **(c)** Box-whisker plot shows Illumina sequencing error profile at repeats of differing length in *repe* genes. Box plot extends from 25th to 75th percentiles, centre line is the median, and whiskers cover the 10th and 90th percentiles (n=1 biologically independent sample). **(d)** Box-whisker plot shows ONT sequencing error profile at repeats of differing length in *repe* genes. Box plot extends from 25th to 75th percentiles, centre line is the median, and whiskers cover the 10th and 90th percentiles (n=1 biologically independent sample). Source data are provided in a Source Data File.

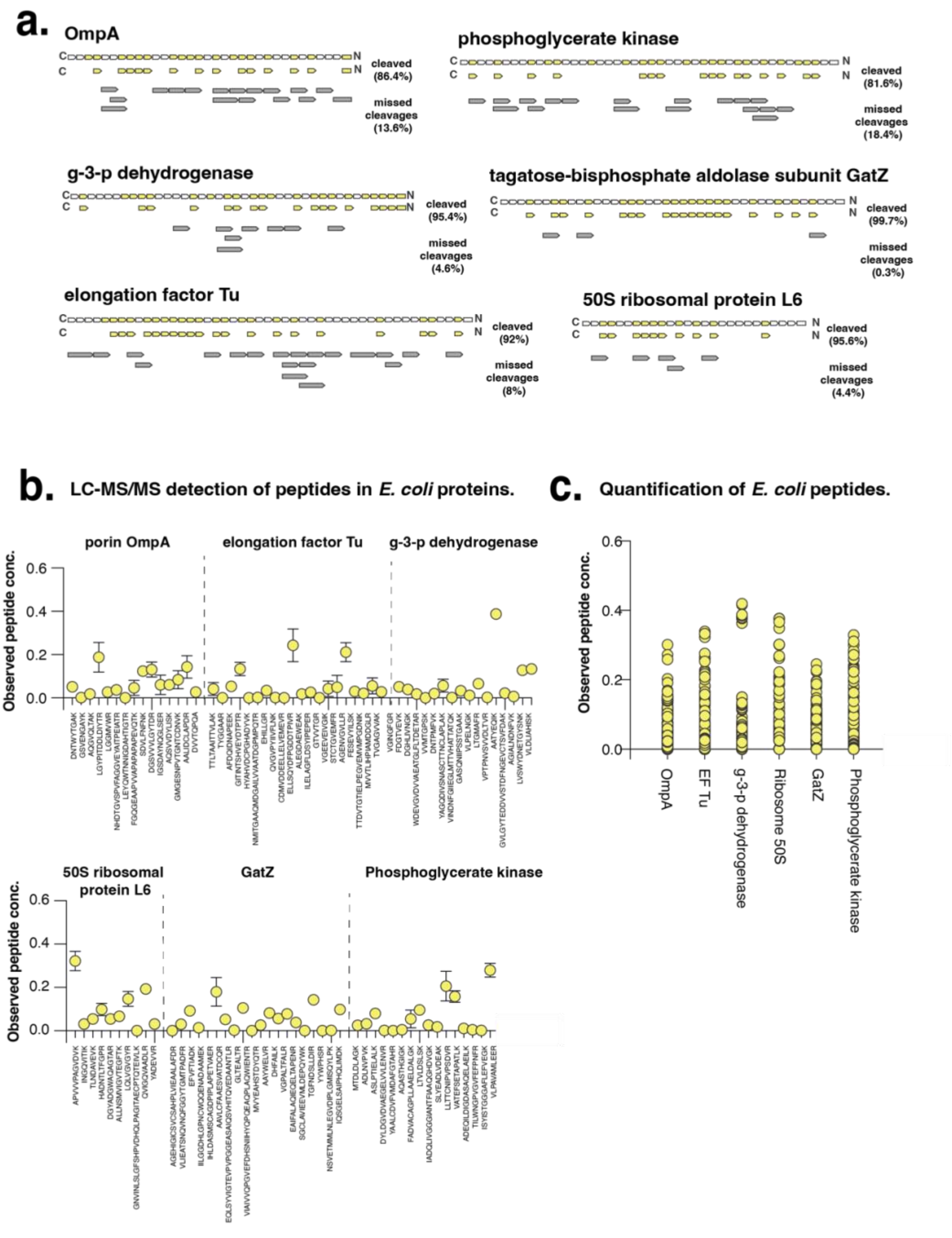

Supplementary Figure S11. Analysis of peptides from abundantly expressed *E. coli* proteins. (a) Proportion of fully cleaved peptides for six abundantly expressed *E. coli* proteins, including OmpA, elongation factor Tu, G-3-p dehydrogenase, 50S ribosomal protein L6, tagatose-bisphosphate aldolase subunit GatZ and phosphoglycerate kinase. For each protein, top row depicts the organisation of peptides theoretically detectable by DIA (yellow = theoretically detectable; white = undetectable), middle row depicts the completely cleaved peptides detected by DIA, and shaded bars in lower lines depict detected partially cleaved peptides. Percentage of fully cleaved and partially cleaved peptides is indicated for each protein. **(b)** Relative quantification of each peptide detected for the six abundantly expressed *E. coli* proteins. Data are presented as mean values +/- SD (n=3 biologically independent samples). **(c)** Measurement of relative abundance for housekeeping *E. coli* proteins (where each peptide is expected to be in equal abundance in replicate (n=3 biologically independent samples). Source data are provided in a Source Data File.

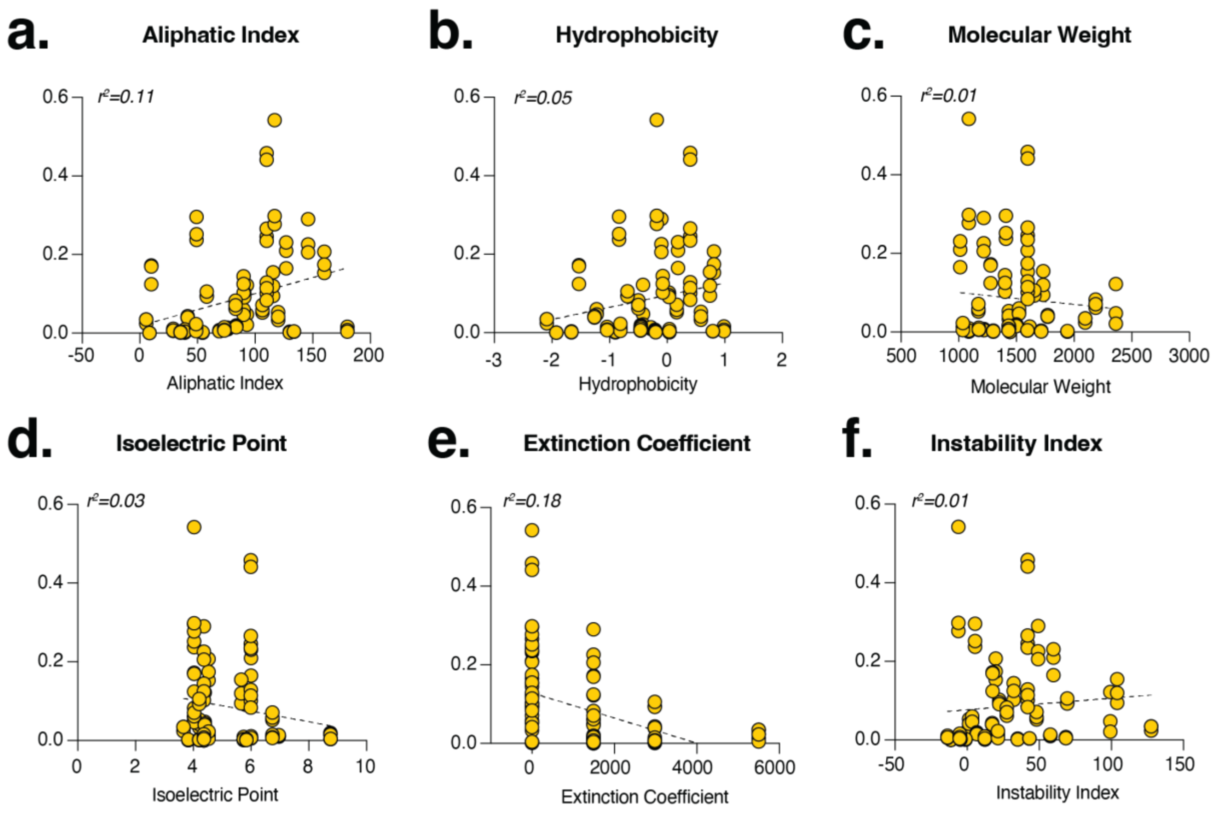

Supplementary Figure S12. Correlation between predicted proco peptide physicochemical properties on peptide quantification. Physicochemical properties were predicted for all proco peptides using Expasy ProtParam including **(a)** Aliphatic Index, **(b)** Hydrophobicity, **(c)** Molecular Weight, **(d)** Isoelectric Point, **(e)** Extinction Coefficient and **(f)** Instability Index. Their values were plotted against relative quantification of each peptide. All peptide quantification values are presented as a proportion of the total detected fully cleaved peptides for each proco protein in each replicate. Source data are provided in a Source Data File.

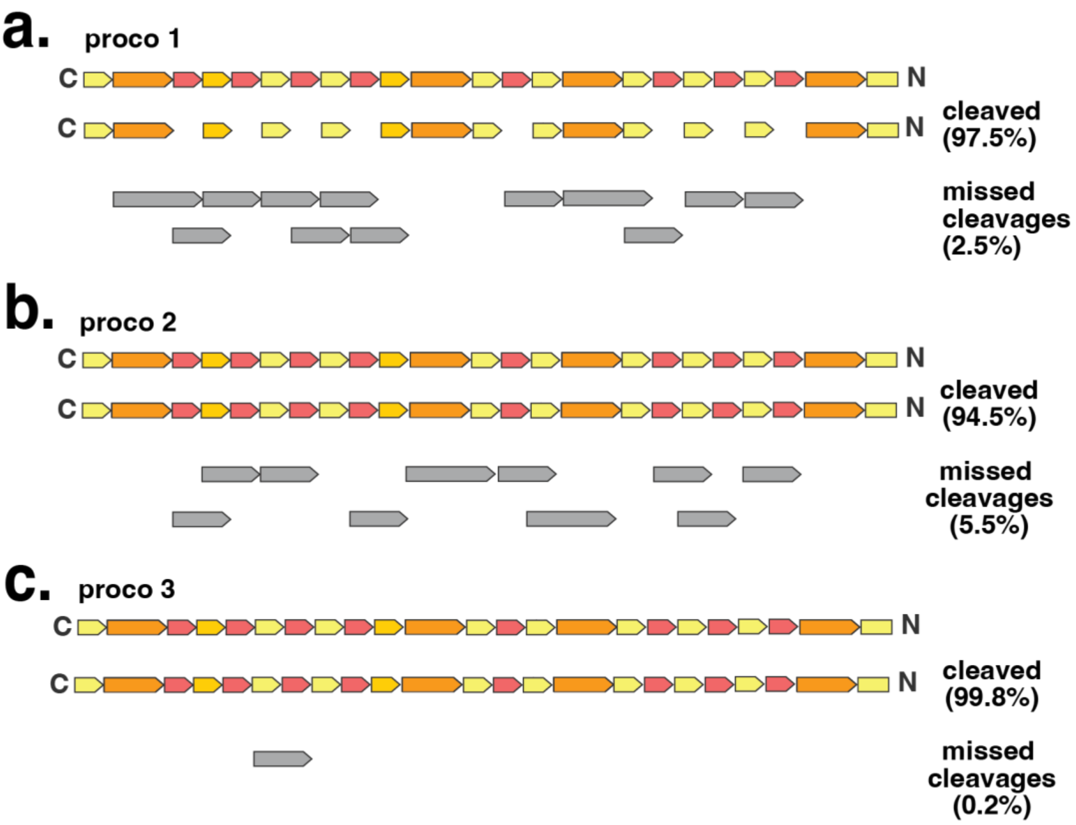

Supplementary Figure S13. Percentage of trypsin cleavage for proco protein. Proportion of complete and missed cleavages for **(a)** proco1, **(b)**, proco2 and **(c)** proco3. For each proco protein, top row depicts the organisation of peptides theoretically detectable by DIA (yellow = 1x, gold = 2x, orange = 4x, red = 8x), middle row depicts the completely cleaved peptides detected by DIA, and shaded bars in lower lines depict detected partially cleaved peptides. Percentage of fully cleaved and partially cleaved peptides is indicated for each protein.

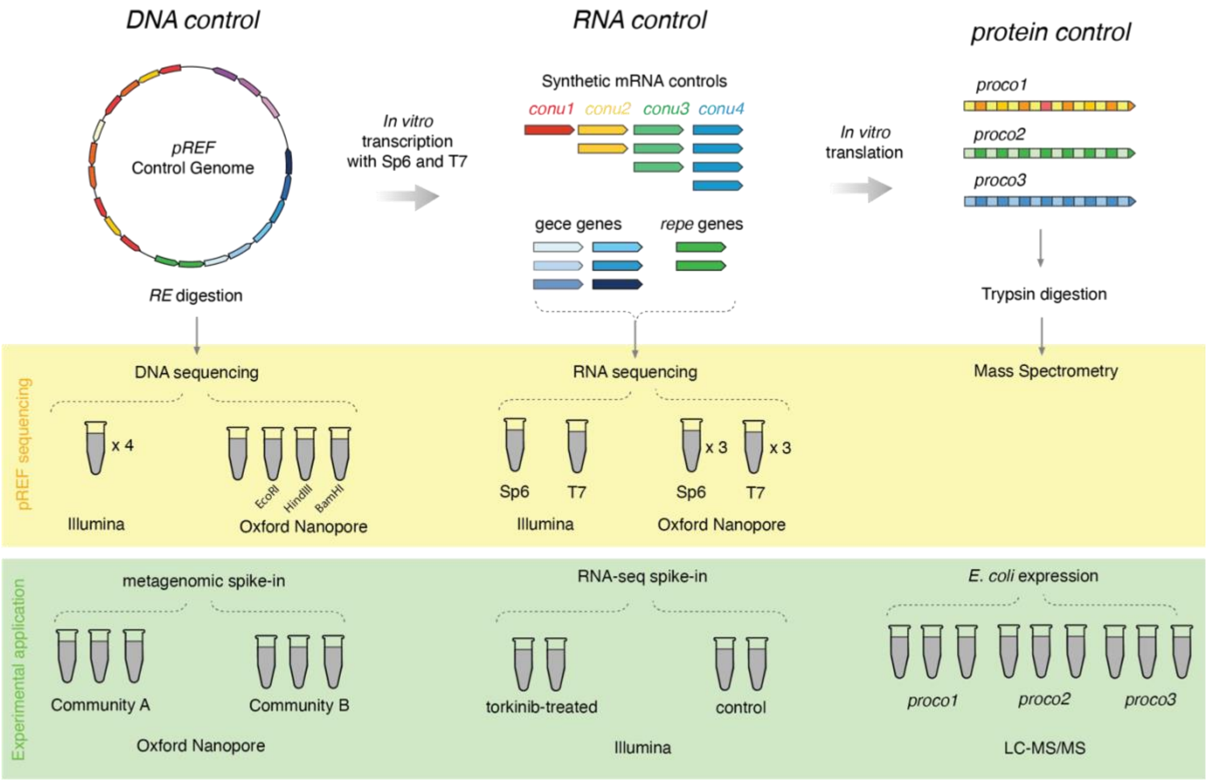

Supplementary Figure S14. Schematic diagram of experiments described in this manuscript. After digestion with different Restriction Enzymes, *pREF* DNA was sequenced on ONT and Illumina platforms. This DNA was used as a spike-in in ONT metagenomics sequencing. *pREF* was then transcribed using T7 and Sp6 polymerase, and sequenced on ONT and Illumina platforms. These RNAs were then used as a spike-in for an Illumina RNA-seq experiment. Last, *pREF* was translated by *E. coli*, and used as controls in a Mass Spectrometry experiment.
