# Supplementary material for: A universal molecular control for DNA, mRNA and protein expression
Source: Nat Commun. 2024 Mar 20;15:2480. doi: 10.1038/s41467-024-46456-9 (PMC10954659; doi:10.1038/s41467-024-46456-9)
Supplement: Supplementary file 2 — Reporting Summary [file 41467_2024_46456_MOESM2_ESM.pdf]

nature portfolio

Corresponding author(s): Timothy Mercer
Last updated by author(s): 2024/02/07

Reporting Summary

Nature Portfolio wishes to improve the reproducibility of the work that we publish. This form provides structure for consistency and transparency in reporting. For further information on Nature Portfolio policies, see our Editorial Policies and the Editorial Policy Checklist.

Statistics

For all statistical analyses, confirm that the following items are present in the figure legend, table legend, main text, or Methods section.

n/a	Confirmed	
☐	☒	The exact sample size (*n*) for each experimental group/condition, given as a discrete number and unit of measurement
☒	☐	A statement on whether measurements were taken from distinct samples or whether the same sample was measured repeatedly
☒	☐	The statistical test(s) used AND whether they are one- or two-sided<br>*Only common tests should be described solely by name; describe more complex techniques in the Methods section.*
☒	☐	A description of all covariates tested
☒	☐	A description of any assumptions or corrections, such as tests of normality and adjustment for multiple comparisons
☐	☒	A full description of the statistical parameters including central tendency (e.g. means) or other basic estimates (e.g. regression coefficient) AND variation (e.g. standard deviation) or associated estimates of uncertainty (e.g. confidence intervals)
☒	☐	For null hypothesis testing, the test statistic (e.g. *F*, *t*, *r*) with confidence intervals, effect sizes, degrees of freedom and *P* value noted<br>*Give P values as exact values whenever suitable.*
☒	☐	For Bayesian analysis, information on the choice of priors and Markov chain Monte Carlo settings
☒	☐	For hierarchical and complex designs, identification of the appropriate level for tests and full reporting of outcomes
☒	☐	Estimates of effect sizes (e.g. Cohen's *d*, Pearson's *r*), indicating how they were calculated

Our web collection on statistics for biologists contains articles on many of the points above.

Software and code

Policy information about availability of computer code

Data collection	*Provide a description of all commercial, open source and custom code used to collect the data in this study, specifying the version used OR state that no software was used.*
Data analysis	All custom scripts and code used during the analysis are available from the GitHub repository: https://zenodo.org/records/10608215<br>Spectronaut (vlS.2.210819.50606)<br>Expasy PeptideCutter (v3.0)<br>Kallisto (v0.46.2)<br>HTSeq (v2.0.l)<br>Jellyfish (v2.2.10)<br>ShortCAKE<br>bowtie2 (v2.4.0)<br>MiniMap2 (v2.17-r941)<br>samtools (vl.9)<br>ONT Albacore Sequencing Pipeline Software (vl.2.6).<br>R (v4.0.2)<br>I Ranges (v2.22.2)<br>edgeR (v3.30.3)<br>RUVg (vl.22.0)

For manuscripts utilizing custom algorithms or software that are central to the research but not yet described in published literature, software must be made available to editors and reviewers. We strongly encourage code deposition in a community repository (e.g. GitHub). See the Nature Portfolio guidelines for submitting code & software for further information.

Data

Policy information about availability of data

All manuscripts must include a data availability statement. This statement should provide the following information, where applicable:

- Accession codes, unique identifiers, or web links for publicly available datasets
- A description of any restrictions on data availability
- For clinical datasets or third party data, please ensure that the statement adheres to our policy

The Next Generation Sequencing (NGS) data generated in this study have been deposited in the SRA database. The gDNA and translated RNA sequencing data were submitted with the PRJNA815898 BioProject Accession Identifier (https://www.ncbi.nlm.nih.gov/sra/PRJNA815898). Metagenomic data were submitted with the PRJNA781348 BioProject Accession Identifier (https://www.ncbi.nlm.nih.gov/sra/PRJNA781348) , and the RNA-seq data were submitted with the PRJNA1073056 BioProject Accession Identifier (https://www.ncbi.nlm.nih.gov/sra/PRJNA1073056). RNA-seq data were annotated based on the GENCODE primary assembly annotation v36 (https://www.gencodegenes.org/human/release_36.html). BAM alignments are available through DRYAD (doi:10.5061/dryad.k0p2ngffn). The Mass Spectrometry DIA data generated in this study have been submitted to the PRIDE database, and are available via ProteomeXchange with identifier PXD035035 (doi:). Source data are provided with this paper.

Research involving human participants, their data, or biological material

Policy information about studies with human participants or human data. See also policy information about sex, gender (identity/presentation), and sexual orientation and race, ethnicity and racism.

Reporting on sex and gender	N/A
Reporting on race, ethnicity, or other socially relevant groupings	N/A
Population characteristics	N/A
Recruitment	N/A
Ethics oversight	N/A

Note that full information on the approval of the study protocol must also be provided in the manuscript.

Field-specific reporting

Please select the one below that is the best fit for your research. If you are not sure, read the appropriate sections before making your selection.

☒ Life sciences ☐ Behavioural & social sciences ☐ Ecological, evolutionary & environmental sciences

For a reference copy of the document with all sections, see nature.com/documents/nr-reporting-summary-flat.pdf

Life sciences study design

All studies must disclose on these points even when the disclosure is negative.

Sample size	Sample size was generally set as n=3 as this is a commonly employed sample size for measuring variability in Next Generating Sequencing data. Most data presented in our manuscript were derived from synthetic samples, removing the biological sources of variability that can lead to the need for increased sample sizes.
Data exclusions	No data excluded
Replication	Sample size was generally set as n=3 as this is a commonly employed sample size for measuring variability in Next Generating Sequencing data. Most data presented in our manuscript were derived from synthetic samples, removing the biological sources of variability that can lead to the need for increased sample sizes. Our chosen level of replication permitted the measurement of technical variability in our samples, and all attempts indicated minimal technical variability.
Randomization	No randomisation was used in our study, but rather, technical variability was limited in our study, through barcoding our libraries and sequencing them together on the same flow cell.
Blinding	No blinding was used in our study, as operator bias was unlikely to impact the results of our study.

Reporting for specific materials, systems and methods

We require information from authors about some types of materials, experimental systems and methods used in many studies. Here, indicate whether each material, system or method listed is relevant to your study. If you are not sure if a list item applies to your research, read the appropriate section before selecting a response.

Materials & experimental systems

n/a	Involved in the study
☒	☐ Antibodies
☐	☒ Eukaryotic cell lines
☒	☐ Palaeontology and archaeology
☒	☐ Animals and other organisms
☒	☐ Clinical data
☒	☐ Dual use research of concern
☒	☐ Plants

Methods

n/a	Involved in the study
☒	☐ ChIP-seq
☒	☐ Flow cytometry
☒	☐ MRI-based neuroimaging

Eukaryotic cell lines

Policy information about cell lines and Sex and Gender in Research

Cell line source(s)	Lung adenocarcinoma AS549 cells
Authentication	Cell lines were not authenticated upon receipt from ATCC
Mycoplasma contamination	Confirm negative mycoplasma contamination
Commonly misidentified lines (See ICLAC register)	None

Plants

Seed stocks	N/A
Novel plant genotypes	N/A
Authentication	N/A
